# The Distribution Characteristics and Potential Risk Assessment of Lead in the Soil of Tieguanyin Tea Plantations in Anxi County, China

**DOI:** 10.3390/toxics12010022

**Published:** 2023-12-25

**Authors:** Yuanyuan Zhan, Qin Zhu, Xiaolin Li, Changwu Tao, Huogui Su, Yuede Wu, Jinshi Lin, Yue Zhang, Yanhe Huang, Fangshi Jiang

**Affiliations:** 1 Jinshan Soil and Water Conservation Research Center, Fujian Agriculture and Forestry University, Fuzhou 350002, China; fafuzhyzyy@163.com (Y.Z.); 18786937827@163.com (Q.Z.); l1175421404@163.com (X.L.); taocw779@163.com (C.T.); linjs18@163.com (J.L.); zhangyue@fafu.edu.cn (Y.Z.); yanhehuang@163.com (Y.H.); 2 Anxi County Soil Fertilizer Technology Extension Station, Quanzhou 362400, China; 13505918807@163.com (H.S.); wuyuede1234@163.com (Y.W.)

**Keywords:** Tieguanyin tea plantation in Anxi County, soil lead, spatial distribution, ecological risk assessment

## Abstract

Assessing the distribution and risks associated with the soil lead content in the Tieguanyin tea plantations of Anxi County is critical, given the county’s significance as the primary Tieguanyin tea production area in Fujian Province. This study examined the distribution characteristics of soil lead in Anxi County’s tea plantations according to the Kriging spatial interpolation of the parameters of the semivariance function of the exponential model. Moreover, the sources of lead content were analyzed, considering geological backgrounds and anthropogenic influences. Ecological risks and the issuance of early warnings were also assessed. The soil lead content in the rocks of the Tieguanyin tea plantations in Anxi County followed the order: andesite > dacite > rhyolite > granite. The soil lead content gradually decreased from the center toward the east and west, forming four distinct north–south parallel zones. High-lead-content areas were identified at the border of Jiandou, Bailai, and Hushang; in the central part of Lutian; and in the southern part of Huqiu. The high levels of soil lead in the tea plantations possibly originated from industrial and mining activities, automobile exhaust, and agricultural activities. The distribution of single-factor pollution indices and potential risk evaluation based on the Soil Environmental Quality Standard, Environmental Technical Conditions for Tea Production Area, and Environmental Technical Conditions for Organic Tea Production Area indicated that the soil in Tieguanyin tea plantations in Anxi County was clean and safe for tea cultivation.

## 1. Introduction

Tea is an important cash crop in China. Its planting areas are widely distributed in the southern provinces, and the yield and quality of tea are closely related to the soil environment. However, China’s industrialization and substantial fertilizer use have impacted tea plantation soils. In recent years, studies have shown that human activities such as industrialization and agricultural fertilization can continuously increase the concentration of potentially toxic elements in tea plantation soil, leading to excessive heavy metal content in the soil, which poses a threat to human ecosystems and health [1,2]. Lead, a common heavy metal, is nonessential to human metabolism and is classified as a toxic substance by the United States Environmental Protection Agency [3]. Excessive intake of lead from tea can lead to health problems such as kidney disease, bone disease, and reduced intelligence quotient [4]. Studies have shown that a linear relationship exists between the heavy metal content of tea and the heavy metal content of the soil [5]. Tea trees thrive in well-drained and acidic soils, ideally within a pH range from 4 to 6.5. Low-pH soil environments pose a heightened risk for lead accumulation, which, in turn, hampers the soil’s microbial population diversity, thereby affecting the ecosystem of the tea plantation soil and hindering the growth and development of tea plants [6]. With the increase in tea planting years and the amount of nitrogen fertilizer input, the tea plantation soil gradually becomes acidified, which leads to the loss of nutrients such as nitrogen and phosphorus and increases the content of effective lead in the soil, exacerbating the lead accumulation in the tea plant [7].

Tieguanyin has a unique “Guanyin rhyme” and a delicate fragrance. When brewed, it yields a rich and long-lasting aroma, earning it the reputation of “seven bubbles with lingering fragrance”. Owing to its excellent quality and unique fragrance, it is renowned both domestically and internationally. Anxi County is the primary Tieguanyin-tea-producing region and the birthplace of the nationally renowned oolong tea. Its unique natural conditions foster excellent tea cultivation, resulting in high-quality products. In recent years, numerous studies have found that tea plantation soils feature a considerably high lead content, which will significantly limit the market of Tieguanyin. The lead content in the tea plantation soils in Anxi County has also garnered widespread concern. Identifying the sources of lead in tea plantations and implementing restrictions to ensure the soil environment’s safety are pivotal for the development of Tieguanyin in Anxi County. Research has found that geological variations primarily dictate the distribution of heavy metals in soil. It has been observed that soils derived from Permian limestone and Cambrian weakly mineralized dolomite exhibit significantly higher heavy metal content than soils developed from Silurian detrital rock [8]. Sun et al. [9] studied the soil lead content of 15 Tieguanyin tea gardens in Anxi County. They discovered that the surface soil lead content (48.0 mg/kg) significantly exceeded the background value, whereas the deep soil lead content (41.5 mg/kg) showed no significant difference from the background value. This suggests that anthropogenic activities have had a more pronounced impact on the surface soil [8]. Cong et al. [10] found that the roots of the Tieguanyin tea plant in Anxi County contained the highest lead content (23.89 mg/kg), primarily originating from the soil. By quantitatively analyzing the potential sources of heavy metals using the Pb–Sr multi-isotope mixing model, they found that agricultural activities contribute the most to the heavy metal content in tea. Zhang et al. [11] studied heavy metals in tea plantations in southern Fujian and found that 63.27% of the sampling points featured a lead content higher than the natural background value, and that the lead content in tea plantation soil is attributable to human activities. Sun et al. [12] found that lead in tea plantation soil mainly originated from parent material and automobile exhaust, while lead in tea mainly originated from automobile exhaust; automobile exhaust accounted for as high as 80% and 60% of the lead contents in new and old leaves, respectively. At present, there has been no comparative study on the distribution, accumulation, and health risk of lead in tea plantation soil in different geologic background areas of Anxi County, and it is of great significance to quantify the soil lead content and spatial distribution in tea plantations for the safe production of tea.

Studies on soil heavy metal pollution status have focused on the distribution and morphology of multiple metals, with less research on the sources of single metals in tea plantation soil. Therefore, in the present study, to reveal the current situation and sources of soil lead contamination in Tieguanyin tea plantations in Anxi County, soils and rocks were collected from the study area. The contamination and sources of lead in tea plantation soil were evaluated via a combination of stepwise linear regression and spatial-analysis-based methods. The objective was to provide a scientific basis for preventing and controlling the lead in tea plantation soil, which is essential for alleviating the ecological impact on tea plantation soil and protecting human health.

## 2. Materials and Methods

### 2.1. Overview of Study Area

The study area is located in Anxi County, Quanzhou City, Fujian Province, China (117°35′–118°17′ E, 24°50′–25°26′ N). Anxi County belongs to the southern and central subtropical marine monsoon climate. Owing to variations in topography and geomorphology, Anxi features distinct climatic characteristics, falling within the southern subtropical zone. The average annual temperature in the eastern area ranges from 19 to 21 °C, with an annual rainfall of 1600 mm. Meanwhile, in the western region, the average annual temperature varies from 16 to 18 °C, with an annual rainfall of 1800 mm. Anxi County is located in the southeastern extension of the Daiyun Mountains, with a terrain sloping from northwest to southeast. The northwestern part of the county features rolling hills, large slopes, and narrow valleys, with an average elevation of over 700 m. The southeastern part of the county features relatively gentle but hilly and mountainous terrain, with an average elevation of <500 m. The soil types in Anxi County mainly include red soil, yellow soil, paddy soil, latosol, calcareous soil, fluvisols, and purplish soil. Currently, the tea planting area in Anxi County spans over 40,000 hm^2^, with a tea yield of 62,000 t and a total tea output value of CNY 19.1 billion [13].

### 2.2. Sample Collection and Processing

A large and representative tea garden in Anxi County was selected for this study. Considering the actual production situation of tea gardens in Anxi County, a Tieguanyin tea garden located within a 10 m distance from a south-facing slope with a soil layer thickness exceeding 1 m was selected. Two to three pieces of unweathered rocks consistent with the soil’s parent rock were collected. After surface impurities were removed from the rocks, they were preserved in self-sealable bags. A total of 95 rock samples were collected, comprising 78 magmatic rocks, 4 metamorphic rocks, and 13 sedimentary rocks. This collection included 12 pieces of andesite, 39 pieces of dacite, 9 pieces of rhyolite, and 18 pieces of granite. The collected rocks were identified and utilized as the rock background for the sampling site. Some of the rocks were ground and sieved through a 200-mesh sieve, then stored in self-sealing bags for the determination of the heavy metal content.

**Figure 1:**
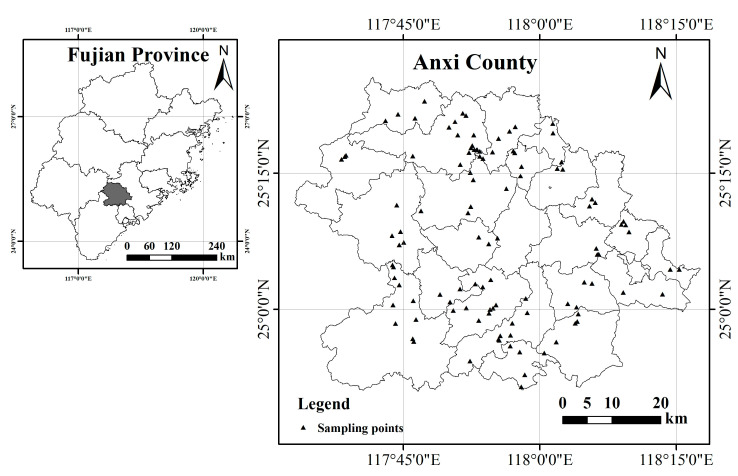
Location of the study area and the sampling points.

Soil samples were collected from selected tea plantations. Composting points and rhizosphere soil were avoided during the collection process. The “S” or plum blossom type 5-point sampling method was employed, with each sampling point comprising more than five sub-samples taken at a depth of 0–20 cm. After mixing the freshly collected soil samples and removing debris, ~1 kg of soil samples was retained, in accordance with the quadratic method. After air drying, the soil samples were passed through 20-mesh and 100-mesh sieves and placed into self-sealing bags with labels for the determination of the soil heavy metals. A total of 109 soil samples were collected and their locations were concurrently recorded using GPS. The distribution of these samples is shown in Figure 1.

### 2.3. Methods of Analysis

The heavy metal elements in the rock samples were determined via X-ray fluorescence spectrometry. The soil lead content was determined through the heat digestion of the soil samples using a mixture of HCl, HF, HNO_3_, and HClO_4_, followed by an analysis using a graphite furnace atomic absorption spectrophotometer. The soil effective lead content was determined via the diethylenetriaminepentaacetic acid–Calcium chloride–triethanolamine combined extraction method and assessed via atomic spectrometry. The soil pH was determined using a pH meter, and the soil organic matter was determined via the potassium dichromate volumetric method. The soil Cu, Mn, Zn, Cd, and Fe contents were determined via inductively coupled plasma-mass spectrometry. The composition of the soil particles was determined using a laser particle sizer (BT-9300ST, Bettersize Instruments Ltd., Dangdong, China). Relevant studies have shown that soil lead is associated with soil Cu, Mn, Zn, and Cd [14,15], and thus these metals were selected for the study. The heavy metals in the rocks of the Tieguanyin tea plantations in Anxi County are shown in the following Table 1.

### 2.4. Potential Ecological Hazard Index

The ecological risk assessment of heavy metals mainly focuses on analyzing the presence and impact of metal element pollution. This study adopts the ecological hazard index method to assess the pollution caused by metal elements. Proposed by Swedish scientist Hakanson [16] in 1980, this method integrates chemistry, biotoxicology, and ecology. It can effectively reflect the impact of individual metal pollutants in specific environments by establishing potential ecological risk indexes for each metal. The potential ecological hazard index method fully considers the toxicity and effectiveness of the metals, and the corresponding toxicity response coefficients of the metals are selected to calculate the potential ecological hazard index E of a single heavy metal element. The calculation formula is as follows:

E = T × C/B(1)

where E is the potential ecological hazard index, C denotes the monitored content, B is the background reference value of the element, and T is the toxicity response factor of the corresponding heavy metal. The ecological hazard level grading and toxicity coefficients of single-factor pollutants are shown in Table 2 [17,18].

### 2.5. Data Processing

SPSS 26, Excel 2010, and OriginPro 2021 were used to analyze the sample data. The spatial distribution map of the soil lead was drawn using GIS10.2 software combined with the Kriging interpolation method.

## 3. Results

### 3.1. Tea Plantation Soil Lead Content

#### 3.1.1. Soil Lead Content in Various Rock Formations

**Figure 2:**
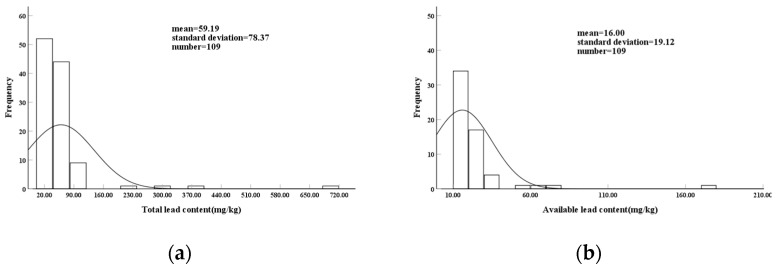
Frequency plot of soil lead content in the study area. (**a**): Histogram of total lead content frequency; (**b**): Histogram of available lead content frequency.

The analysis of the soil lead content in tea plantations across Anxi County revealed average levels of 59.19 mg/kg for the total soil lead and 16.00 mg/kg for effective lead (Figure 2). The lead content in the soil developed from different types of rocks in Anxi County varied. The ranges of soil lead content in the soil derived from the three major rock types were 14.90–700.60 mg/kg for magmatic rocks, 26.90–45.90 mg/kg for metamorphic rocks, and 17.00–53.10 mg/kg for sedimentary rocks. Specifically, within the magmatic rock category, the ranges of soil lead content were as follows: andesite: 21.10–700.60 mg/kg, rhyolite: 15.70–308.50 mg/kg, dacite: 15.70–364.70 mg/kg, and granite: 14.90–118.20 mg/kg. The mean values of soil lead content in the rocks were as follows: magmatic rock (64.70 mg/kg) > metamorphic rock (32.64 mg/kg) > sedimentary rock (30.88 mg/kg). The ranking based on the magnitude of lead content within magmatic rocks was as follows: andesite (99.88 mg/kg) > dacite (62.06 mg/kg) > rhyolite (60.95 mg/kg) > granite (50.32 mg/kg). The coefficients of variation (CVs) for the soil lead content in the three major rock types in Anxi County were as follows: magmatic rock (130.87%) > sedimentary rock (32.06%) > metamorphic rock (23.95%). Specifically, within the magmatic rock category, the CVs for different rocks were as follows: andesite (182.13%) > rhyolite (117.45%) > dacite (93.81%) > granite (52.57%) (Table 3).

A comparative analysis of the soil lead content in different rock types in Anxi County was conducted using the national soil elemental background values by Wei [19] and the soil elemental background values from Fujian Tieguanyin tea plantations obtained by Zhou [20] as control references. The average soil lead content values in the three major rock types were lower than the soil elemental background values of the Fujian Tieguanyin tea plantation. However, among these, only andesite exhibited a higher soil lead content than the soil elemental background values of the Fujian Tieguanyin tea plantation. The average content of the heavy metal lead in the soil from the three major rock types exceeded the national background value for soil elements (Table 4).

#### 3.1.2. Soil Lead Content of Tea Plantations in Different Townships

A statistical analysis was conducted on the soil lead content in tea plantations across various townships of Anxi County (Table 5). The contents of lead in the tea plantation soils in areas such as Hushang (230.95 mg/kg), Lutian (148.98 mg/kg), Huqiu (82.25 mg/kg), Bailai (71.68 mg/kg), and Kuidou (70.05 mg/kg) exceeded the soil environmental quality standard limit (70 mg/kg), showing a decreasing trend from north to south. The soil lead content in all township tea plantations was below the standard environmental limit for tea-producing areas (250 mg/kg). In addition to the high content of available lead (66.02 mg/kg) in the tea plantation soil of Hushang, the available lead content in the other towns was lower than 30 mg/kg. The CV of the lead content in the tea plantation soil exceeded 50% in several areas, such as Hushang (135.64%), Huqiu (112.50%), Lutian (98.78%), Guanqiao (57.63%), Jiandou (52.73%), and other towns (50.08%). Hushang, Huqiu, and Lutian exhibited larger coefficients of variation, which corresponded to higher lead contents. The CVs of the soil available lead content in tea plantations in towns such as Hushang (110.79%), Lutian (90.41%), Jiandou (86.17%), Huqiu (79.14%), Guanqiao (61.85%), other towns (60.07%), Longjuan (56.38%), and Daping (51.12%) exceeded 50%. The CVs of lead in the tea plantation soil in Anxi County revealed that the lead content is not uniformly distributed and is significantly affected by external disturbances.

The national soil elemental background values presented in Wei Fusheng’s article [19] and the soil elemental background values specific to Fujian Tieguanyin tea plantations by Zhou Guohua [20] were used as control background values for the comparative analysis of the average soil lead content in tea plantations across Anxi County. The average lead contents in the soil of the tea plantations in the Huqiu, Kuidou, Lutian, Bailai, and Hushang townships exceeded the soil element background values of the Fujian Tieguanyin tea plantation, with increases of 17.25, 5.05, 83.98, 6.68, and 165.95 mg/kg, respectively. The average lead contents in the soil of the tea gardens in Daping, Guanqiao, Longmen, Penglai, Xiping, Gande, Jiandou, Jingu, Changkeng, Futian, Longjuan, Taozhou, Xianghua, and other townships were lower than the background value of the soil element in the Tieguanyin tea plantations of Fujian. These areas showed decreases of 14.43, 24.12, 3.87, 20.60, 26.56, 25.73, 0.73, 0.93, 17.08, 43.67, 39.32, 34.25, 28.93, and 12.18 mg/kg, respectively. All townships in Anxi County, except for Futian, exhibited a higher tea plantation soil lead content than the national background values (Table 6).

#### 3.1.3. Spatial Distribution of Soil Lead in Tea Plantation

The sample data were fitted via geostatistical methods, using the semi-variance function, and the optimal model was selected. The soil lead content in the study area conformed to the exponential model, and the determination coefficients of the semi-variance function model were all small, indicating that their spatial distribution was relatively dispersed. The nugget effect was used to represent the proportion of the spatial variation in relation to the total variation, thereby serving as a measure for assessing the magnitude of spatial correlation among the study variables. A ratio below 0.25 indicates a relatively strong spatial autocorrelation, while a ratio between 0.25 and 0.75 indicates a moderate spatial autocorrelation. A ratio exceeding 0.75 signifies a weak spatial autocorrelation. Range is another important parameter of the semi-variance function. It indicates the extent of spatial autocorrelation effects within a certain scale. Numerous studies have shown that, the closer the Residual Sum of Squares value is to 0, the better the model fit of the semi-variance function and the shorter the range. The block basis ratios for the soil lead and available lead content were 0.13 and 0.10, respectively, with range values of 4260.00 m and 2490.00 m. These values suggest that variations in the lead content were influenced by both structural and stochastic factors, indicating a relatively strong spatial distribution (Table 7).

**Figure 3:**
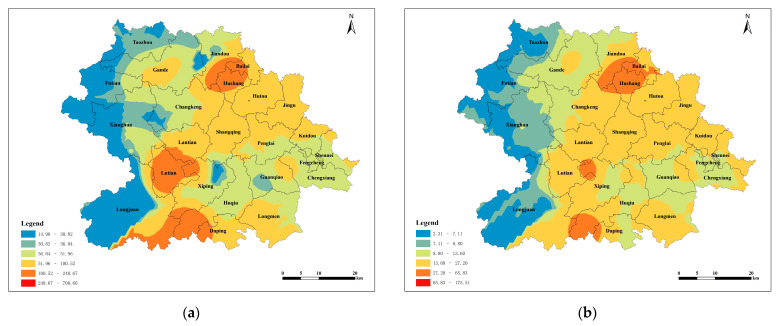
Spatial distribution of lead in Tieguanyin tea plantation soils. (**a**): Spatial distribution of lead in tea plantation soils; and (**b**): spatial distribution of available lead in tea plantation soils.

According to the spatial interpolation of the parameters of the semivariance function of the exponential model, the spatial distribution of the soil lead content in the tea plantations in Anxi County was obtained. The findings revealed a gradual decline in the soil lead content from the county’s central region toward the east and west, resulting in the formation of four distinct and parallel distribution zones oriented in a north–south direction. A high lead content occurred at the borders of Jiandou, Bailai, and Hushang; the central part of Lutian; and the southern region of Huqiu. The distribution of soil available lead content in tea plantations across Anxi County was consistent with the distribution of lead content (Figure 3).

### 3.2. Ecological Risk Evaluation of Lead in Tea Plantation Soil

#### 3.2.1. Evaluation Based on the Soil Environmental Quality Standard

**Figure 4:**
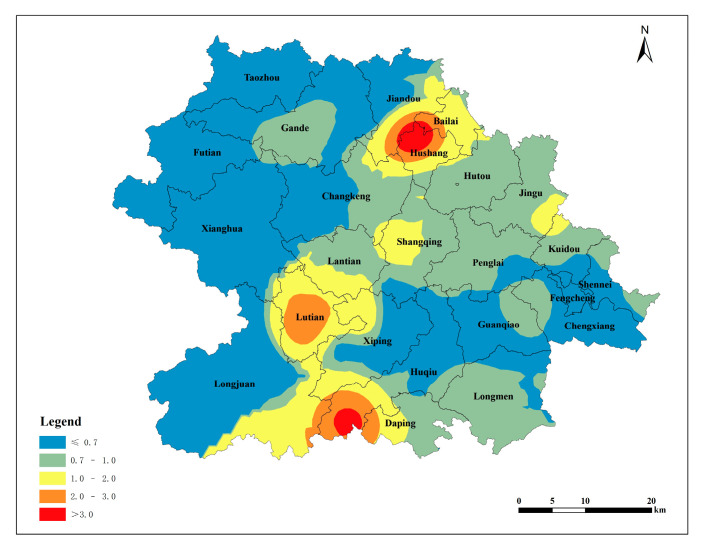
Spatial distribution of individual pollution index of lead in tea plantation soils (The soil environmental quality standard was taken as the basis for evaluation).

The single pollution index was calculated using the measured data of the soil lead content in Anxi County’s tea plantations and the soil pH values, in accordance with the land quality geochemical evaluation specification. The soil environmental quality standard [21] was taken as the basis for evaluation. The spatial distribution map was obtained via Kriging interpolation based on the single pollution index. Heavily polluted areas were identified in the southern part of Huqiu, the central part of Lutian, and the northern part of Hushang. The pollution index gradually decreased from the source area to the surrounding area, indicating a decline in the soil lead content in the tea plantation with an increasing distance from the source area (Figure 4). According to the distribution of the single-factor pollution index based on the soil environmental quality standard (Table 8), 47.37% of the tea plantation soil lead in Anxi County is categorized as clean soil, 31.58% belongs to slight pollution, 10.53% belongs to mild pollution, 5.26% belongs to moderate pollution, and 5.26% belongs to heavy pollution.

#### 3.2.2. Evaluation Based on the Environmental and Technical Conditions of Tea Production Areas

**Figure 5:**
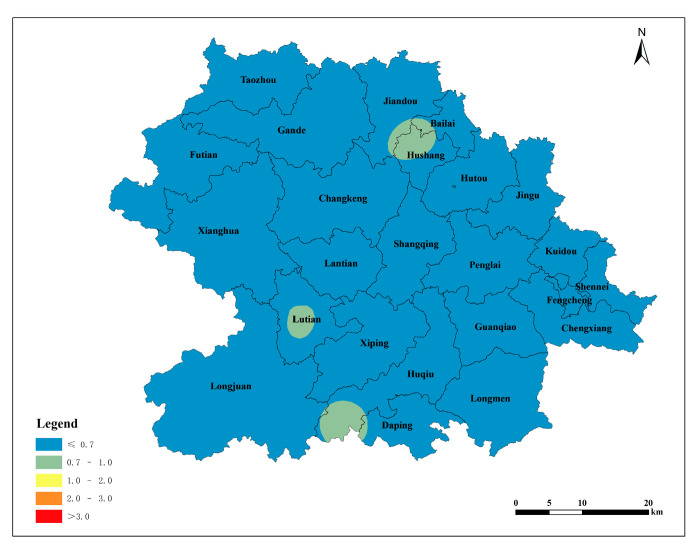
Spatial distribution of individual pollution index of lead in tea plantation soils (The environmental technical conditions of tea origin was taken as the basis for evaluation).

The single pollution index was calculated using the measured data of the lead content in the tea plantation soil in Anxi County and the soil pH values, with the environmental technical conditions of tea origin [22] as the evaluation basis. The spatial distribution map was obtained via Kriging interpolation based on the single pollution index. The northern part of Hushang, the central part of Lutian, and the southern part of Huqiu exhibited second-level pollution, categorized as slight pollution. Other townships in Anxi County displayed clean soil conditions. This indicates that, by the standards of the environmental and technical conditions of tea origin, the soil in Anxi County’s Tieguanyin tea plantations is generally clean and safe. The vast majority of soils are suitable for the production of non-pollution tea (Figure 5). According to the distribution of the single-factor pollution index based on the environmental technical conditions of tea origin (Table 8), 94.74% of the lead in the tea garden soil in Anxi County belongs to clean soil, and 5.26% belongs to slight pollution.

#### 3.2.3. Evaluation Based on the Environmental and Technical Conditions of Organic Tea Production Areas

**Figure 6:**
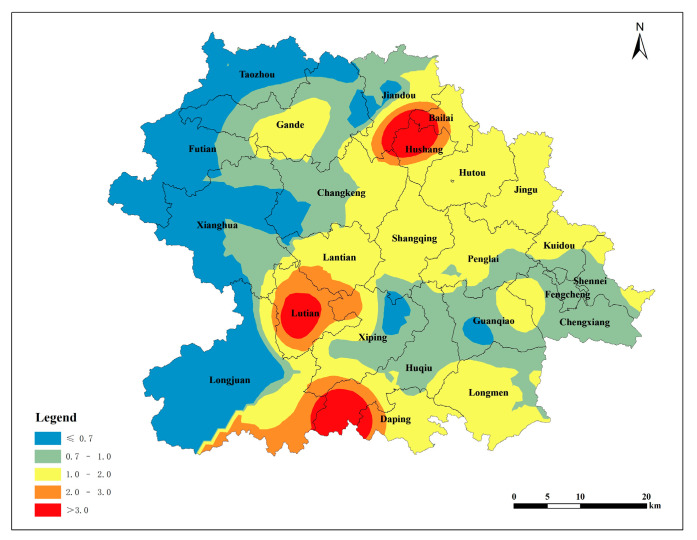
Spatial distribution of individual pollution index of lead in tea plantation soils(The environmental conditions of organic tea production was taken as the basis for evaluation).

The single pollution index was calculated using the measured data of the soil lead content in the tea gardens in Anxi County and the soil pH value, with the environmental conditions of organic tea production [23] as the evaluation basis. The spatial distribution map was obtained via Kriging interpolation based on the single pollution index. The pollution level in the northern part of Hushang, the central part of Lutian, and the southern part of Huqiu reached the fifth level, corresponding to heavy pollution. Areas with the fourth level of pollution were distributed near fifth-level-pollution source areas (Figure 6). The terrain in Anxi County slopes from northwest to southeast, and the primary source areas are mainly concentrated in three regions: the northern part of Hushang, the central part of Lutian, and the southern part of Huqiu. The pollution spreads from the source areas to the lower southeast direction, and the northwest of Anxi County is less affected by the source areas owing to its higher terrain. According to the distribution of the single-factor pollution index derived from the environmental conditions of organic tea production areas (Table 8), the lead content in the tea garden soil in Anxi County is categorized as follows: 15.79% as clean soil, 31.58% as slight pollution, 42.11% as mild pollution, 5.26% as moderate pollution, and 5.26% as heavy pollution.

#### 3.2.4. Evaluation of Potential Ecological Risk

**Figure 7:**
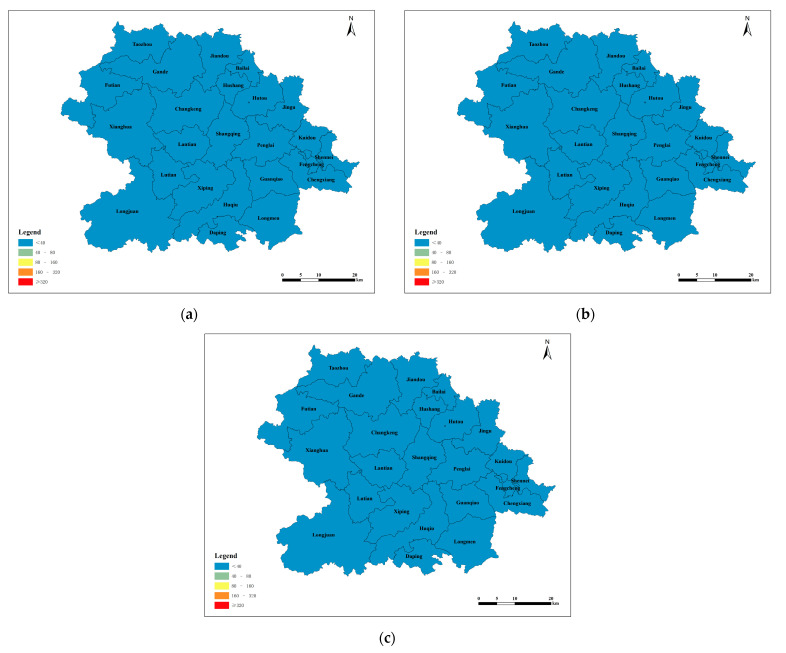
Spatial distribution of potential ecological hazard index of soil lead content in tea plantation. (**a**): The soil environmental quality standard is used as the background value; (**b**): the background value is based on the environmental and technical conditions of tea producing areas; and (**c**): the background value is based on the environmental and technical conditions of organic tea production areas.

The potential ecological hazard index of the soil lead in the Tieguanyin tea plantations of Anxi County was calculated using a formula that considers the measured soil lead content and the background value and toxicity coefficient of lead. Then, the spatial distribution of the potential ecological hazard index of the soil lead in the Tieguanyin tea plantations in Anxi County was obtained via Kriging interpolation (Figure 7). The spatial distribution maps of the potential ecological hazard index, generated using the background values of Soil Environmental Quality Standard, Environmental Technical Conditions for Tea Production Area, and Environmental Technical Conditions for Organic Tea Production Area, indicated that the soil lead pollution in the Tieguanyin tea plantations in Anxi County was at a low level, posing no potential risk of pollution.

### 3.3. Relationship between Soil Lead Content and Factors in Tea Plantation

**Figure 8:**
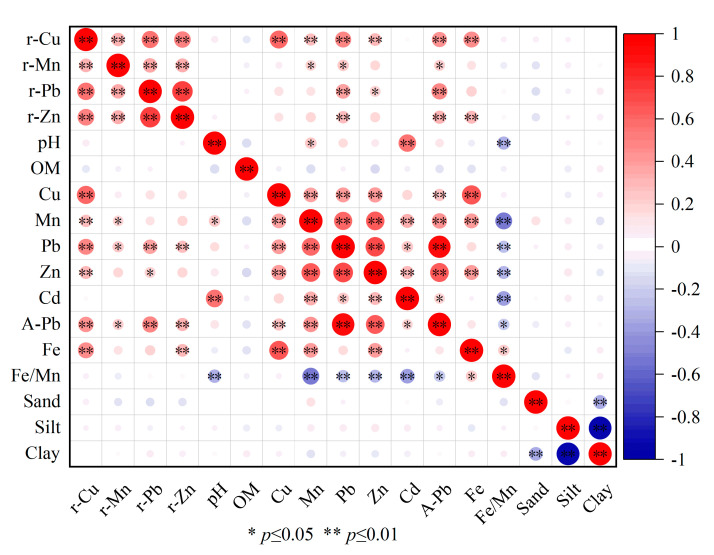
Correlation analysis of lead content in soil with rocks, soil elements and physicochemical properties.

The correlation between the lead content in the soil of Tieguanyin tea gardens and rocks, soil elements, and physical and chemical properties was analyzed. The results showed a significant correlation between the lead content and rock Mn. Moreover, the soil lead exhibited a significant correlation with rock lead, Cu, and Zn, indicating a close relationship between soil lead and rocks. Additionally, a significant correlation existed between soil lead and Cd, and a highly significant correlation existed between soil lead and Cu, Mn, and Zn, indicating that soil lead may have the same source pathway as Cu, Mn, Zn, and Cd. A highly significant correlation existed between the soil lead and soil available lead, both of which had no correlation with the soil pH, organic matter, soil Fe, sand, silt, and clay. A significant correlation existed between the soil available lead and Fe/Mn, and a highly significant correlation existed between the soil lead and Fe/Mn. The correlation between the soil available lead and other indicators is consistent with that of soil lead (Figure 8).

**Figure 9:**
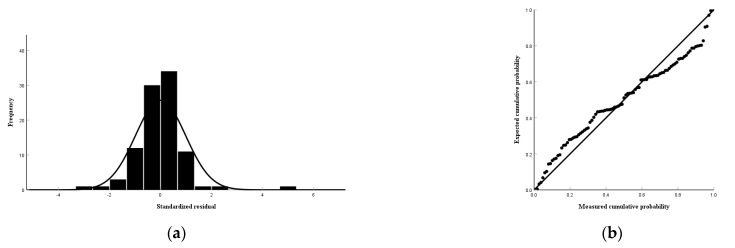
Standard residual histograms (**a**) and normal P−P plot (**b**) of the multiple linear regression equation for soil lead.

**Figure 10:**
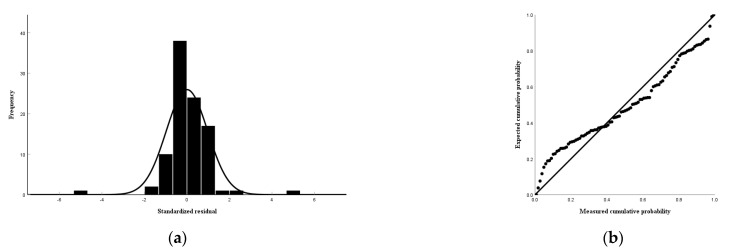
Standard residual histograms (**a**) and normal P−P plot (**b**) of the multiple linear regression equation for soil available lead.

According to the close relationship between the soil lead, rock, and soil indicators, the soil lead and available lead of the Tieguanyin tea plantations in Anxi County were selected as dependent variables, and the closely related indicators were used as the independent variables to establish multiple linear regression equations. The F-value of the third model was 44.541, and the *p*-value was 0.00, which is less than 0.01, indicating that the independent variables in the model jointly had a highly significant effect on the dependent variable; that is, the rock Cu, soil Zn, and Mn had a highly significant effect on the soil lead content (Table 9). The standardized coefficient of the soil Zn was greater than that of the rock Cu and soil Mn, indicating that the soil Zn had a greater effect on the soil lead (Table 10). The F-value of the fifth model was 151.770, and the *p*-value was 0.00, which is less than 0.01, indicating that the independent variables in the model jointly had a highly significant effect on the dependent variable; that is, the soil lead, Mn, Cu, Zn, and rock lead had a highly significant effect on the soil available lead content (Table 11). Furthermore, the absolute value of the standardized coefficient of the soil lead was greater than those of the rock lead, soil Mn, lead, Zn, and Cu, indicating that the soil lead had the greatest effect on the soil available lead (Table 12). The variance inflation factors of the soil lead and available lead were less than 10, and the covariance relationship between the variables was not significant (Table 10 and Table 12). According to the above, the optimal fitting multiple linear regression equations for soil lead and available lead were obtained (Table 13). The regression standardized residuals of the soil lead and available lead followed a normal distribution, which indicates that the multiple linear regression equations were well-fitted (Figure 9 and Figure 10).

Rock lead samples, which accounted for 74.31% of the total samples, exhibited lower lead levels than the soil lead. The ratio of rock lead to soil lead was 0.90 (Table 14), suggesting that the soil lead in Tieguanyin tea plantations in Anxi County was not solely a result of parent rock weathering. There might be additional lead introduced due to anthropogenic activities. The ratio of soil available lead to soil lead was 0.27, indicating the reactivity of soil lead in the Tieguanyin tea plantations of Anxi County. This portion of lead can easily migrate from soil to tea trees, become absorbed by the tea trees, and accumulate in the tea. Upon consumption of the tea, this lead enters the human body, posing a hidden health risk.

## 4. Discussion

### 4.1. Distribution Characteristics and Source Analysis of Soil Lead Content in Tea Plantation

Soil is formed through the long-term weathering of rocks, and its elemental composition and content largely inherit the geochemical characteristics of the parent rock. Most of the elements in soil are derived from the parent rock [24]. Through geochemical and chronological studies on the Carboniferous magmatic rock of the Sauer Mountains in northwestern Junggar, China, Weng et al. [25] found that potassium feldspar granite exhibited high levels of potassium (K) and lead, clear I-type affinity, and high potassium–calcium alkalinity. Some related studies have also found that the abnormal lead content in granite originates from the crustal composition [26,27]. Ahankoub et al. [28] found significant lead enrichment in all samples during their study of magmatic rocks in western Iran. During their study of tea gardens in the metamorphic region of Guizhou Province, Li et al. [29] found that some soil samples exceeded the heavy metal standards, but this did not impact the growth and development of the tea. Additionally, the tea plant did not show enrichment of As, Cr, Hg, and lead from the soil. According to the geological survey results of Anxi County, tuff lava is predominantly found in the regions of Gande, Xianghua, Bailai, Jingu, Kuidou, Penglai, Lutian, Huqiu, Xiping, and southern Longmen. Granite is mainly distributed in Hushang, Changkeng, Lantian, Shangqing, Guanqiao, Longjuan, and Longmen. Sandstone is predominantly located in Futian, northern Taozhou, central Xianghua, southern Taozhou, and southern Longjuan. Magmatic rock accounts for 89.9% of the county’s area, and the soil weathering process associated with magmatic rock serves as a significant source of soil lead accumulation. In this study, the soil derived from magmatic rock exhibited the highest lead content (64.7 mg/kg) and the most pronounced variability (130.87%), aligning with previous research findings and the geological background of Anxi County. Moreover, the geological composition of Lutian and the southern region of Huqiu predominantly consisted of rhyolitic tuff lava, which continuously contributed lead to the soil in Lutian and the southern part of Huqiu during the soil weathering process.

The magnitude of soil lead content depends on the local geological background and is strongly influenced by factors such as industrial production, agricultural fertilization, and traffic exhaust [30]. Cao et al. [31] conducted a risk evaluation of tea plantation soil in Anxi County and found that the average concentration of soil lead was 1.24 times the background value. The elevated lead content primarily originated from anthropogenic sources related to agricultural and industrial activities. Lead is a signature element of motor vehicle pollution. The friction between the tires of a moving vehicle and the ground produces Zn [32]. This aligns with the highly significant correlation observed in this study between the two elements, suggesting a possible link between abnormal lead levels in tea plantation soil and automobile exhaust. Atmospheric deposition from coal combustion in industrial and mining industries leads to the enrichment of lead in soil [33]. The mining industry is more developed near the convergence of Jiandou, Bailai, and Hushang in Anxi County, which is consistent with the observed high soil lead content distribution in this study. Long-term directed fertilization has a certain effect on soil lead and Cd content [34]. Both soil lead and Cd exhibit similarities. The southern tea plantations in Anxi County are primarily core tea plantation areas, and the elevated soil lead content in this region might be linked to prolonged fertilization practices. The research results suggest that a portion of the soil lead in tea plantations in Anxi County originates from the soil parent rock, while a larger portion may result from automobile exhaust, atmospheric deposition, and agricultural activities. There appears to be a higher likelihood of contribution from automobile exhaust. Tea plantations in Anxi County should be located as far away from roads and mines as possible to reduce the impact of anthropogenic activities on heavy metal accumulation in tea plantation soil. To improve the soil properties and mitigate the ecological risks associated with heavy metals in tea plantation soil, measures tailored to soil conditions and tea tree growth, such as the application of organic fertilizers [35] or amendments [36], can be implemented on susceptible plantations.

### 4.2. Potential Risks and Early Warning concerning Soil Lead in Tea Plantation

Ecological risk warning involves a qualitative analysis that combines quantitative evaluation values with pollution grading. This approach, to a certain extent, reflects the pollution status of the experimental site and issues a warning, to provide a basis for management actions. In this study, the soil lead content of the Tieguanyin tea plantations of Anxi County was higher than the national soil element background value, indicating that the tea plantation soil in Anxi County features a certain amount of lead pollution. The evaluation and early warning of heavy metal pollution in soil, coupled with in-depth research on contaminated soil and assessments of potential risk situations in polluted areas, holds important practical significance for the governance and protection of these areas. In this study, the qualitative analysis of tea plantation soil in Anxi County was based on the evaluation of the Soil Environmental Quality Standard, Environmental Technical Conditions of Tea Production Areas, and Environmental Technical Conditions of Organic Tea Production Areas. The potential ecological pollution index of lead in the soil of the Tieguanyin tea plantations of Anxi County was determined to be at a low level, indicating the absence of a potential pollution risk.

The heavy metals in tea garden soil mainly exist in several forms: water-soluble, exchangeable, organic-bound, oxidized iron and manganese, carbonate, and residual. The bioavailability of the water-soluble and exchangeable forms is high, and they are greatly affected by the soil pH [37,38]. The available state of soil heavy metals is the portion that plants can absorb and accumulate. Evaluating this state is complex, owing to various influencing factors, including plant species, root exudation, soil organic matter, and soil pH [39,40,41]. In this study, the ratio of soil available lead to the total lead amount was 0.27; this ratio helps to assess the amount of lead available in the soil. A higher utilization rate signifies increased lead activity and easier absorption by tea trees. The distribution order of soil lead and available lead in each township’s tea plantation in Anxi County varies, possibly owing to differences in soil pH across the townships’ tea plantations. The planting times of tea plantations vary across the townships of Anxi County, with southern plantations having longer durations. The long-term cultivation of tea plants has resulted in the accumulation of organic matter in tea plantation soil and a decrease in soil pH. This decrease has caused an increase in the water-soluble and exchangeable lead content absorbed by tea plants [37]. The distribution and migration of lead in soil and tea are affected by soil organic matter and redox processes [42]. Soil iron, manganese, and pH play pivotal roles in altering redox conditions and organic chemical processes [43]. In the present study, the soil available lead was significantly correlated with the soil Mn and Fe/Mn, and no correlation existed between the soil available lead and the soil Fe or pH. This indicates that the distribution of lead in soil is affected by the geochemical behavior of Mn [44,45]. Therefore, the management of soil lead pollution in tea production areas in Anxi County can focus on altering the structure of soil lead, reducing the levels of available lead, and mitigating the ecological risks posed by soil lead on the plant growth environment.

## 5. Conclusions

(1) The lead content in soils developed from the three major rock types was generally lower than the background soil element value in the Fujian Tieguanyin tea plantation. Only the average lead content in soils derived from andesite in magmatic rocks exceeded the background value of soil elements in the Fujian Tieguanyin tea plantation. The average soil lead content developed by these rock types is higher than the national background value.
(2) The soil lead content in Tieguanyin tea plantations within the townships of Huqiu, Kuidou, Lutian, Bailai, and Hushang exceeded the background value of soil elements in Tieguanyin tea plantations in Fujian. Except for Futian, other townships of Anxi County’s Tieguanyin tea plantations exhibited a soil lead content higher than the national background value of soil elements. The distribution of soil lead in Anxi County’s Tieguanyin tea plantations was uneven and significantly impacted by external factors.
(3) The soil lead content of the Tieguanyin tea plantations of Anxi County gradually declined from the center to the east and west, forming four distinct parallel distribution zones in the north–south direction. High-lead-content areas occurred at the convergence of Jiandou, Bailai, and Hushang; in Lutian’s central region; and in the southern part of Huqiu. The distribution of available lead content in the tea plantation soil of Anxi County was consistent with the lead content distribution.
(4) The spatial distribution map of potential ecological hazard indexes, based on the background values of the Soil Environmental Quality Standard, Environmental Technical Conditions for Tea Production Area, and Environmental Technical Conditions for Organic Tea Production Area, indicated that the soil lead pollution in the Tieguanyin tea plantations of Anxi County was at a low level, posing no risk of potential pollution. Part of the soil lead in the Tieguanyin tea plantations of Anxi County originates from the soil parent rock, but a larger portion is attributed to automobile exhaust, atmospheric deposition, and agricultural activities, with automobile exhaust being the more likely source.

## Figures and Tables

**Table 1:** Descriptive statistics of heavy metal content in rocks and soils of Tieguanyin tea plantations in Anxi County (mg/kg).

Type	Norm	Mean	SD	CV	Min	Max
Rock	Cu	9.93	15.07	1.52	0.30	82.30
	Mn	750.84	1197.67	1.60	78.00	9741.00
	Pb	53.33	86.52	1.62	8.10	640.60
	Zn	84.47	126.77	1.50	8.90	1006.50
Soil	Cu	12.38	9.90	0.80	0.30	56.7
	Mn	403.38	377.38	0.94	65.00	2356.00
	Zn	71.68	37.28	0.52	20.90	262.80
	Cd	0.05	0.04	0.66	0.003	0.18
	Fe	3.38	1.71	0.51	1.18	10.64

The cadmium content of rock was not tested; thus, it is not presented in this paper.

**Table 2:** Classification of ecological hazards and toxicity coefficient of the single factor pollutants.

Ecological Hazard Level	Low	Medium	High	Higher	Extremely High	Toxicity Factor
E	<40	40–80	80–160	160–320	≥320	5

**Table 3:** Lead content in soils with different rock development in Anxi County.

Rock Type		Number	Mean (mg/kg)	SD	Min (mg/kg)	Max (mg/kg)	CV
magmatic rock	andesite	13	99.88	181.90	21.10	700.60	182.13%
	rhyolite	15	60.95	71.58	15.70	308.50	117.45%
	dacite	43	62.06	58.22	15.70	364.70	93.81%
	granite	20	50.32	26.45	14.90	118.20	52.57%
Three major rock type	magmatic	91	64.70	84.67	14.90	700.60	130.87%
	metamorphic	5	32.64	7.82	26.90	45.90	23.95%
	sedimentary	13	30.88	9.90	17.00	53.10	32.06%

**Table 4:** Comparison of lead content and background values in soils developed from different rocks in Anxi County.

Rock Type		A	B	C	AB	AC
magmatic rock	andesite	99.88	65.00	23.30	34.88	76.58
	rhyolite	60.95	65.00	23.30	−4.05	37.65
	dacite	62.06	65.00	23.30	−2.94	38.76
	granite	50.32	65.00	23.30	−14.69	27.02
three major rock type	magmatic	64.70	65.00	23.30	−0.30	41.40
	metamorphic	32.64	65.00	23.30	−32.36	9.34
	sedimentary	30.88	65.00	23.30	−34.12	7.58

A represents the mean value of lead content in different rock development soil in Anxi County; B and C represent the background values of soil elements in Fujian Tieguanyin Tea Plantation and China, respectively; and AB and AC represent the difference between the mean value of lead content in different rock development soil in Anxi County and the respective background values, respectively.

**Table 5:** Determination results of lead content in tea garden soil in Anxi County.

Study Area		Number	Lead (mg/kg)		Available Lead (mg/kg)	
			Mean	CV	Mean	CV
Anxi County	Daping	4	50.58 ± 20.30	40.14%	11.13 ± 5.69	51.12%
	Guanqiao	5	40.88 ± 23.56	57.63%	10.53 ± 6.51	61.85%
	Huqiu	8	82.25 ± 92.53	112.50%	20.54 ± 16.26	79.14%
	Kuidou	4	70.05 ± 20.82	29.72%	22.43 ± 9.54	42.54%
	Longmen	3	61.13 ± 12.41	20.29%	15.64 ± 5.99	38.31%
	Penglai	3	44.40 ± 12.70	28.60%	12.72 ± 2.62	20.64%
	Xiping	9	38.44 ± 12.58	32.72%	13.60 ± 6.05	44.51%
	Lutian	5	148.98 ± 147.16	98.78%	28.67 ± 25.92	90.41%
	Bailai	4	71.68 ± 16.10	22.46%	19.97 ± 6.84	34.27%
	Gande	15	39.27 ± 14.98	38.15%	10.58 ± 4.75	44.84%
	Jiandou	6	64.27 ± 33.89	52.73%	23.70 ± 20.42	86.17%
	Jingu	3	64.07 ± 5.08	7.93%	13.22 ± 2.53	19.17%
	Changkeng	5	47.92 ± 14.41	30.06%	14.54 ± 6.62	45.55%
	Futian	3	21.33 ± 8.33	39.04%	5.51 ± 2.19	39.79%
	Hushang	4	230.95 ± 313.27	135.64%	66.02 ± 73.14	110.79%
	Longjuan	10	25.68 ± 7.06	27.48%	6.07 ± 3.42	56.38%
	Taozhou	4	30.75 ± 3.86	12.57%	6.19 ± 2.33	37.61%
	Xianghua	6	36.07 ± 7.88	21.85%	8.46 ± 2.01	23.78%
	Other townships	8	52.83 ± 26.45	50.08%	16.53 ± 9.93	60.07%
Entire study area		109	61.31 ± 86.06	140.37%	16.52 ± 20.85	126.60%

Townships with too few sampling points were aggregated into other townships for analysis; 2 in Shennei, 2 in Chengxiang, 2 in Lantian, 1 in Hutou, and 1 in Shangqing.

**Table 6:** Comparison of soil lead content and background values in tea plantations of various towns in Anxi County.

Townships	A	B	C	AB	AC
Daping	50.58	65.00	23.30	−14.43	27.28
Guanqiao	40.88	65.00	23.30	−24.12	17.58
Huqiu	82.25	65.00	23.30	17.25	58.95
Kuidou	70.05	65.00	23.30	5.05	46.75
Longmen	61.13	65.00	23.30	−3.87	37.83
Penglai	44.40	65.00	23.30	−20.60	21.10
Xiping	38.44	65.00	23.30	−26.56	15.14
Lutian	148.98	65.00	23.30	83.98	125.68
Bailai	71.68	65.00	23.30	6.68	48.38
Gande	39.27	65.00	23.30	−25.73	15.97
Jiandou	64.27	65.00	23.30	−0.73	40.97
Jingu	64.07	65.00	23.30	−0.93	40.77
Changkeng	47.92	65.00	23.30	−17.08	24.62
Futian	21.33	65.00	23.30	−43.67	−1.97
Hushang	230.95	65.00	23.30	165.95	207.65
Longjuan	25.68	65.00	23.30	−39.32	2.38
Taozhou	30.75	65.00	23.30	−34.25	7.45
Xianghua	36.07	65.00	23.30	−28.93	12.77
Other townships	52.83	65.00	23.30	−12.18	29.53

A represents the average value of soil lead content in tea gardens in each township of Anxi County; B and C represent the background value of soil element in Fujian Tieguanyin tea plantation and China, respectively; and AB and AC represent the difference between the average value of soil lead content in tea plantation in each township of Anxi County and each background value, respectively.

**Table 7:** Semi-variance function model and parameters for spatial interpolation of lead in tea plantation soil.

Norm	Model	Nugget	Sill	Nugget/Sill	Range (m)	R^2^	RSS
total lead	index	0.05	0.39	0.13	4260.00	0.33	0.036
available lead	index	0.05	0.49	0.10	2490.00	0.15	0.037

**Table 8:** Distribution of single-factor pollution index for the three evaluation criteria in Anxi County. Unit: %.

Study Area	Evaluation Criteria	Standard Limit Value	*p* ≤ 0.7	0.7 < *p* ≤ 1.0	1.0 < *p* ≤ 2.0	2.0 < *p* ≤ 3.0	*p* > 3.0
Anxi County	Environmental quality standards for soil	70	47.37	31.58	10.53	5.26	5.26
	Environmental technical conditions for tea production areas	250	94.74	5.26	0.00	0.00	0.00
	Environmental technical conditions for organic tea production areas	50	15.79	31.58	42.11	5.26	5.26

**Table 9:** Analysis of variance table for regression equation of soil lead content ^a^.

Model		Sum of Square	Degrees of Freedom	Mean Square	F	Significance
1	regression	321,330.737	1	321,330.737	89.799	0.000 ^b^
	residual	332,786.816	93	3578.353		
	total	654,117.553	94			
2	regression	372,504.551	2	186,252.275	60.847	0.000 ^c^
	residual	281,613.003	92	3061.011		
	total	654,117.553	94			
3	regression	389,119.208	3	129,706.403	44.541	0.000 ^d^
	residual	264,998.345	91	2912.070		
	total	654,117.553	94			

^a^ Dependent variable lead; ^b^ predictor variable: (constant), Zn; ^c^ predictor variable: (constant), Zn, rock Cu; and ^d^ predictor variable: (constant), Zn, rock Cu, Mn.

**Table 10:** Stepwise regression results of soil lead content.

Model		Unstandardized Coefficient		Standardization Coefficient	T	Significance	Collinearity Statistics	
		Beta	Standard Error				Tolerance	VIF
1	(Constant)	−46.823	13.011		−3.599	0.001		
	Zn	1.490	0.157	0.701	9.476	0.000	1.000	1.000
2	(Constant)	−49.190	12.048		−4.083	0.000		
	Zn	1.301	0.153	0.612	8.529	0.000	0.909	1.101
	Rock Cu	1.624	0.397	0.293	4.089	0.000	0.909	1.101
3	(Constant)	−46.891	11.790		−3.977	0.000		
	Zn	1.028	0.188	0.483	5.474	0.000	0.571	1.752
	Rock Cu	1.506	0.391	0.272	3.855	0.000	0.894	1.119
	Mn	0.044	0.018	0.210	2.389	0.019	0.576	1.735

**Table 11:** Analysis of variance for regression equation of available lead content ^a^ in soil.

Model		Sum of Square	Degrees of Freedom	Mean Square	F	Significance
1	regression	32,888.392	1	32,888.392	518.171	0.000 ^b^
	residual	5902.729	93	63.470		
	total	38,791.122	94			
2	regression	33,676.203	2	16,838.101	302.860	0.000 ^c^
	residual	5114.919	92	55.597		
	total	38,791.122	94			
3	regression	34,172.926	3	11,390.975	224.455	0.000 ^d^
	residual	4618.196	91	50.749		
	total	38,791.122	94			
4	regression	34,461.925	4	8615.481	179.108	0.000 ^e^
	residual	4329.197	90	48.102		
	total	38,791.122	94			
5	regression	34,719.160	5	6943.832	151.770	0.000 ^f^
	residual	4071.962	89	45.752		
	total	38,791.122	94			

^a^ Dependent variable: available lead; ^b^ predictor variable: (constant),lead; ^c^ predictor variable: (constant), lead, Mn; ^d^ Predictor variable: (constant),lead, Mn, rock lead; ^e^ predictor variable: (constant), lead, Mn, rock lead, Cu; and ^f^ predictor variable: (constant), lead, Mn, rock lead, Cu, Zn.

**Table 12:** Stepwise regression results of soil available lead content.

Model		Unstandardized Coefficient		Standardization Coefficient	T	Significance	Collinearity Statistics	
		Beta	Standard Error				Tolerance	VIF
1	(Constant)	2.766	1.020		2.712	0.008		
	lead	0.224	0.010	0.921	22.763	0.000	1.000	1.000
2	(Constant)	5.047	1.130		4.464	0.000		
	lead	0.250	0.012	1.027	21.727	0.000	0.641	1.560
	Mn	−0.009	0.002	−0.178	−3.764	0.000	0.641	1.560
3	(Constant)	4.006	1.130		3.545	0.001		
	lead	0.236	0.012	0.968	19.745	0.000	0.544	1.837
	Mn	−0.008	0.002	−0.160	−3.525	0.001	0.631	1.584
	rock lead	0.029	0.009	0.124	3.129	0.002	0.831	1.203
4	(Constant)	5.474	1.252		4.370	0.000		
	lead	0.241	0.012	0.991	20.368	0.000	0.523	1.910
	Mn	−0.007	0.002	−0.137	−3.023	0.003	0.603	1.658
	rock lead	0.030	0.009	0.126	3.262	0.002	0.831	1.203
	Cu	−0.190	0.078	−0.096	−2.451	0.016	0.805	1.242
5	(Constant)	2.781	1.668		1.667	0.099		
	lead	0.227	0.013	0.931	17.273	0.000	0.406	2.462
	Mn	−0.009	0.002	−0.177	−3.742	0.000	0.526	1.901
	rock lead	0.031	0.009	0.130	3.451	0.001	0.829	1.206
	Cu	−0.215	0.076	−0.109	−2.813	0.006	0.790	1.266
	Zn	0.065	0.027	0.125	2.371	0.020	0.422	2.370

**Table 13:** Optimal multivariate linear fitting equations for soil lead content.

Dependent Variable	Multiple Linear Regression Equation	R^2^	Significance
lead	Y = 1.028 X_5_ + 1.506 X_6_ + 0.044 X_2_ − 46.891	0.595	0.000
available lead	Y = 0.277 X_1_ − 0.009 X_2_ + 0.031 X_3_ − 0.215 X_4_ + 0.065 X_5_ + 2.781	0.895	0.000

X_1_ is soil lead; X_2_ is soil Mn; X_3_ is rock lead; X_4_ is soil Cu; X_5_ is soil Zn; and X_6_ is rock Cu.

**Table 14:** Comparison of rock and soil lead content in tea plantation.

Norm	<1	=1	>1	Mean Value Ratio
Lead of rock/Lead of soil	74.31%	0.00%	25.69%	0.90
Available lead of soil/Lead of soil	100.00%	0.00%	0.00%	0.27

## Data Availability

Data are contained within the article.
